# Education Research: What Medical Students Value in Neurology Residents: A Qualitative Study to Inform Resident as Teacher Curricula

**DOI:** 10.1212/NE9.0000000000200135

**Published:** 2024-06-10

**Authors:** Harry W. Sutherland, Jeremy J. Moeller, Sara M. Schaefer

**Affiliations:** From the Department of Neurology, Yale School of Medicine, New Haven, CT.

## Abstract

**Background and Objectives:**

Residents are responsible for much of the formal and informal teaching of neurology clerkship medical students. High-quality resident teachers can enhance clerkship satisfaction, decrease neurophobia, and increase specialty interest. To train such residents, some institutions have developed resident as teacher (RAT) curricula. Existing RAT curricula are highly variable, partly because of our limited understanding of medical student attitudes and expectations regarding the qualities and skills of effective resident teachers. We sought to identify important themes in resident teaching, based on qualitative analysis of written evaluations by students, to better inform future RAT curricula in neurology.

**Methods:**

Clerkship student evaluations of residents from 2012 to 2023 at a single institution were collected and anonymized. The narrative comments were thematically coded using conventional content analysis in an iterative process of reconciliation and recoding. Randomly selected evaluations were analyzed in batches of 50 at a time until thematic saturation was achieved.

**Results:**

A total of 200 evaluations yielded 6 themes with 27 subthemes: (1) Work-based learning, teaching and assessment: “sets expectations,” “student involvement in care,” “student autonomy over care,” “helps students prepare/practice,” “gives feedback,” “mentorship and coaching,” and “challenges students”; (2) Attitudes as teacher: “likes to teach,” “made time to teach,” “inspirational/fun,” “patience,” “approachability,” and “learner-centric”; (3) Learning environment: “safety” and “clear communication”; (4) Role modeling: “knowledge,” “skills,” “attitudes,” and “leadership”; (5) Content of teaching: “clinical skills,” “medical knowledge,” “nonmedical topics,” and “directed to further learning”; and (6) Context of teaching: “bedside/in exam room,” “attending rounds,” “in workflow,” and “break for teaching.” The most prevalent subthemes were “student involvement in care,” “gives feedback,” “safety,” “made time to teach,” and “approachability.”

**Discussion:**

In their written evaluations of neurology residents, medical students identified many attributes, skills, and methods that led to a positive learning experience. Many of these themes highlighted the importance of residents facilitating work-based learning, cultivating the learning environment, and serving as role models rather than formal teaching activities alone. We provide recommendations for further RAT curricular development informed by these results. Using these findings, we further illustrate how residents influence the tripartite interaction between the learner, their subject, and their environment seen in existing learning theories.

## Introduction

When he created the first clerkships for medical students in the late 19th century, William Osler expected that medical students would learn best through bedside teaching rounds and by practicing under the supervision of residents.^1^ This is congruent with our current understanding that learners learn best through experience.^2^ In most modern academic medical centers, hospital residents are most directly involved in the day-to-day operations of the inpatient wards. Clerkship students, therefore, have extensive contact with residents throughout their undergraduate training on the wards, with up to 25% of residents' time being dedicated to direct student engagement.^3^ Because of this close relationship, hospital residents function simultaneously as clinicians in training and as teachers of medical students—facilitating student learning through “legitimate peripheral participation” in the practice and community of medicine.^4^ In 1 survey, medical students responded that as much as half of their knowledge was gained during rounds with residents.^5^ In this way, residents are central to shaping the clerkship experience and demonstrating the practice and culture of medicine—what has been deemed the “hidden curriculum.”^6^

Students enjoy their relationships with residents with several studies demonstrating greater clerkship satisfaction when there is greater contact with excellent resident teachers.^7-10^ Students appreciate that residents are uniquely positioned to teach medical students as “near-peers” with fresh insight into their experience.^11^ In fact, it has been demonstrated in simulation-based,^12-14^ small-group,^15^ and lecture settings^16^ that residents may be more effective and preferred by medical students compared with attending physicians. In addition to intentional and explicit teaching, much of resident teaching is informal and includes role modeling behaviors or referencing the literature on rounds.^17^

Despite the large role residents play in medical student education, most residents are not provided with much formal training on effective teaching techniques. In addition, residents may feel unable to teach because of the competing demands of patient care. Because of this, most residents may not feel that they have the necessary time or tools to teach “properly.”^18,19^ To provide residents with the requisite skills, a growing number of training programs have developed “resident-as-teacher” (RAT) curricula. These RAT curricula in and outside of neurology have widely variable focuses and scopes with respect to microskills covered and encounter setting.

Little is known about what medical students themselves regard as the qualities and skills of an effective resident teacher. Most available publications on the principles of effective medical teaching date before 2000 and a large proportion, especially the more seminal papers, use self and peer evaluation of expert teacher practices rather than learner perspectives. While these insights are highly valuable, they may not fit the needs and expectations of current clerkship students in a learner-centered way. A large number of investigations are also targeted to ambulatory care where the teaching skills may not be entirely transferrable to the inpatient setting. When learner perspectives are assessed, they tend to be of faculty rather than resident teaching. Applying these findings to resident teaching for RAT curricular development may not be entirely valid because residents tend to cover slightly different material than attending teachers and have different stylistic approaches to teaching given their unique relationship to medical students.^20^ Notably, none of these findings have come from the field of neurology.

This study aims to provide a deeper appreciation of medical student attitudes and expectations with respect to qualities and skills of effective resident teachers. We propose that resultant lessons could guide further development of neurology RAT curricula that focus on more learner-centric microskills with the goal to create more effective resident educators. Possible secondary but complementary outcomes to updated RAT curricula informed by this study would be increased clerkship satisfaction, comfort with neurology patients and problems, and specialty interest. Our study will aim to understand this medical student perspective through the qualitative analysis of narrative comments on clerkship teaching evaluation forms.

## Methods

### Educational Context

A typical medical school clerkship class at Yale School of Medicine consists of approximately 100 students. Medical students participate in a required 4-week neurology clerkship during the 12-month core clerkship experience which spans from January of their second year through December of year 3. The Neurology clerkship is housed within a 12-week interdisciplinary block which also includes 8 weeks of Internal Medicine. The 4 weeks of Neurology are divided into two 2-week rotations which may be completed on the General, Stroke, Consult, Neuro Intensive Care Unit, Pediatric, or Veterans Affairs (VA) inpatient services, or in various subspecialty clinics. The VA rotation includes about 50% dedicated time in outpatient general and subspecialty neurology clinics with residents in addition to inpatient time. Most medical students spend much or all of their clerkship time on adult neurology teams.

From March through June 2020, medical student clinical participation in clerkship responsibilities and didactics was entirely remote because of the coronavirus disease 2019 (COVID-19) pandemic. The joint internal medicine-neurology clerkship was shortened to 10 weeks until January 2022 but the neurology portion always remained 4 weeks.

On the inpatient services, residents are expected to supervise students. Mandatory resident training includes three 1-hour noon conference didactics on bedside teaching, feedback, and learning theory. Additional optional resident training is available through education journal clubs hosted by the residency program, institution-wide enrichment programs, bimonthly sessions through the Yale School of Medicine Center for Medical Education, and through an official Neurology Clinician-Educator Distinction pathway. All residents receive written feedback on their teaching performance during semi-annual performance reviews with the program director or associate program director.

### Student Evaluations of Resident Teaching

At the end of their core Neurology clerkship, students are asked to identify which residents they worked most closely with so those residents may accurately assess their clinical knowledge, skills, and attitudes. Students fill out a reciprocal evaluation of their selected residents. Students can view resident evaluations in the online learning management system, but residents only see aggregated evaluations when at least 5 have been completed, and at their semi-annual reviews to minimize the risk of loss of student anonymity. The current student evaluation of a resident form, in use since 2015, instructs the student to “please evaluate the teaching by this physician” on a Likert scale of 1–5 (1 = poor, 2 = fair, 3 = good, 4 = very good, 5 = excellent). The students are then asked to enter free text with the following prompt: “please type any comments you wish to make about this physician.” Prior or alternative teaching forms used either the same prompt or simply requested “teaching comments:” or “comments:” Comments are a required component of the evaluation form.

Anonymized student evaluations of residents from 2012 to 2023 were downloaded from the learning management system (MedHub) by author J.J.M. (residency program director). Evaluations of former residents currently at this institution were not available for inclusion. These evaluations were then provided to author S.M.S. (associate residency program director) who copied the narrative comments into a spreadsheet and redacted resident names before sharing with author HS (resident) for analysis.

### Qualitative Analysis

Two authors (S.M.S. and H.W.S.) reviewed the student narrative comments on resident evaluation forms and coded for different subthemes that appeared within the text using conventional content analysis.^21^ Conventional content analysis was chosen because of the open-ended nature of the evaluation prompt and because it is not dependent upon existing theory or the authors' pretest hypotheses.^22^ As discussed above, existing literature on residents as teachers is largely derived from a prior generation of students and outside the field of neurology so may not have been entirely applicable to ground this work in.

Evaluations were sorted in random order and then analyzed in “batches” of 50 evaluations at a time. The 2 coding authors independently reviewed each batch of 50 evaluations to generate subthemes. The 2 authors met to reconcile their subthemes, organize them into larger themes, and compile and/or update a codebook after each batch. After each update, previously analyzed comments were re-evaluated using the new codebook. This iterative process continued until thematic saturation was achieved and both coding authors agreed no new codes arose from the analysis of an entire batch of evaluations. The overall coding scheme was reviewed with a third author not involved in the coding process itself (J.J.M.) at each stage to ensure that the themes/subthemes and organization were credible.

Author H.W.S. was never provided with the names of residents whose evaluations were included in the analysis. No other special actions were taken either in the coding or reconciliation processes owing to his status as a resident. Several techniques were used to ensure there was appropriate reflexivity during data analysis, including constant comparative analysis, memo keeping during the coding stage, and explicit discussions between the authors about the effect of their roles as neurology educators. Reflexivity will be discussed further in the Discussion section.

### Standard Protocol Approvals, Registrations, and Patient Consents

This study protocol was approved by the Yale Human Research Protection Program Institutional Review Board (FWA00002571). This ethics board waived the requirement for participant consent in this retrospective analysis.

### Data Availability

Anonymized resident evaluations and/or coding workbooks will be made available by request from any qualified investigator. This data will be available for a period of at least 2 years after publication.

## Results

Of all available medical student evaluations of resident teachers, a total of 200 were analyzed before saturation. These spanned from 2015 to 2023. One hundred sixty-one used the current evaluation form and an additional 30 were from a prior form which used the same prompt. Seven evaluations were from a form soliciting “teaching comments:” and 2 were in response to a box for “comments:” Twenty-seven subthemes were identified as a result of this analysis. These subthemes were organized into 6 themes. Please see Table 1 for a listing of themes, subthemes, and illustrative quotations.

**Table 1:** Six Themes and 27 Subthemes Identified by Textual Analysis of Narrative Comments in Medical Student Evaluations of Resident Teachers

Theme	Subtheme	Illustrative quotation(s)
1. Work-based learning, teaching and assessment	1a. Sets expectations	“From the first day he set clear expectations…”
	1b. Student involvement in care	“…allowed us to have ownership over our patients, such as calling consults, communicating with nurses, and helping with discharge documents.”
	1c. Student autonomy over care	“He would always let me enter the room first and perform the physical exam…”
	1d. Helps students prepare/practice	“He always checked in with the medical student to briefly go through plans before rounds…”
	1e. Gives feedback	“[Resident] offered much fair and encouraging feedback.”
	1f. Mentorship and coaching	“a great mentor… always looking for opportunities for me to learn…”
	1g. Challenges students	“She had high standards for our presentations, assessments, and plans… but also made us feel safe and supported.”
2. Attitudes as teacher	2a. Likes to teach	“She loves teaching medical students and greatly improves the student experience with her words of wisdom and enthusiasm.”
	2b. Made time to teach	“Despite a busy census and tight schedule, he invariably made time to teach and provide feedback.”
	2c. Inspirational, fun	“[Resident's] passion for neurology was clear, and this made me feel more excited about the service and our cases, too.”
	2d. Patience	“He was very patient with us during our first rotation.”
	2e. Approachability	“I always felt comfortable going to [resident] with questions.”
	2f. Learner-centric	“…considered my specific interests and career goals.”
3. Learning environment	3a. Psychological safety	“He created a fun and positive environment for all team members, which allowed me to feel comfortable asking questions.”
	3b. Clear communication	“[Resident] is a great communicator and I consistently knew what to do, where to be, what my role was on the team, etc.”
4. Role modeling	4a. Knowledge	“She is clearly very knowledgeable and knows her patients well.”
	4b. Skills	“He did a wonderful job connecting with patients in such an intense environment, and that's not always easy to do.”
	4c. Attitudes	“[Resident] is so clearly passionate about his work, caring about his patients”
	4d. Leadership	“Simply observing him run the service… provided a great deal of learning…”
5. Content of Teaching	5a. Clinical skills	“She would teach me specific neuro exam maneuvers that were relevant.”
	5b. Medical knowledge	“She took time to show students the interesting neurology cases she's seen in the past.”
	5c. Nonmedical	“…really appreciated life advice on work-life balance and managing personal life through residency!”
	5d. Directed to further reading	“…offered resources to explore the field of pediatric neurology.”
6. Context of teaching	6a. Bedside/In exam room	“Did a great job with presenting teaching points during encounters and made sure something was always learned.”
	6b. Attending rounds	“He used to take time out to explain things to the student and expand on the conversation that was being had during rounds.”
	6c. In workflow	“…encouraging me to learn things alongside him”
	6d. Break for teaching	“Though he has a ton of work to do himself, he always makes himself available to our questions and even initiates teaching sessions himself!”

The first theme included comments on work-based learning, teaching, and assessment. The first subtheme was expectation setting which largely consisted of orientation to the service and student roles. The next 2 concerned student involvement in and autonomy over patient care. Some recurrent examples included assigning suitable teaching cases, providing students with updates throughout the day, and valuing student contributions. Students liked observing residents perform patient care tasks they themselves could not (e.g., procedures, delicate family meetings) and appreciated chances to lead where appropriate (e.g., bedside rounds/clinic visits, calling a consult, prepping a note). Helping students prepare or practice was the next subtheme with students highlighting patient presentation rehearsals and discussing clinical reasoning before rounds. Providing formative and summative feedback was a prominent subtheme for medical students. In fact, several of the rare negative comments centered around lack of feedback—reinforcing the desire of medical students to be provided with constructive ways to improve. Mentorship and coaching, whether broadly at a career level or narrowly at an encounter level was another subtheme. Finally, medical students appreciated being challenged to excel by their residents. This took the form of high standards and encouraging greater independence in patient encounters and medical decision making.

Theme 2 focused on specific attitudes of resident teachers. Students appreciated residents who were fond of teaching and made efforts to find time to teach despite their demanding clinical responsibilities. They responded well to residents who were inspiring or made the subject and rotation fun. They lauded residents who were patient and approachable. Students also commented on certain learner-centric behaviors like soliciting student goals and being adaptive to the needs and desires of the student.

Theme 3 concerned the learning environment. Medical students made note of whether they felt psychologically safe and supported working with their clinical teams. Clear communication skills of residents were also featured, especially when it came to directing students.

Theme 4 comprised comments on residents' role modeling behaviors of competent clinicians. Medical students often commented on how knowledgeable residents were about their specialty and the patients they carried. Skills ranging from diagnostic, to procedural, to interpersonal were often described. Attitudes of residents with respect to patient care and professionalism within a multidisciplinary care team were featured. Leadership qualities of senior residents also arose. There were many praises for residents who served as aspirational models for students to follow.

In theme 5, students commented on the content of teaching they received from their residents. Medical skills (e.g., physical examination skills, imaging interpretation) and medical knowledge (e.g., illness scripts) predominated. Residents also directed learners to additional resources for further reading. Nonmedical topics also appeared, including specialty selection, wellness and work-life balance, tips for systems-based practice, and maintaining personal identity.

Theme 6 reflected the variety of settings in which learning took place. Some educational activities occurred within protected spaces for dedicated teaching sessions. Others were situated within the clinical context including at the bedside or in the examination room, on attending rounds, or otherwise in the daily workflow of completing tasks directly related to patient care.

The most prevalent subthemes coded within student evaluations were “student involvement in care” (subtheme 1b), “gives feedback” (1e), “safety” (3a), “made time to teach” (2b), and “approachability” (2e). Overall, student evaluations of residents were very positive. Negative comments were a relative rarity and focused on a desire for more of the positive qualities already shared above. These desires included wanting more teaching generally, more bedside teaching (subtheme 6a) specifically, more feedback (1e), and to feel more included within the team (1b or 3a).

## Discussion

In their written evaluations of neurology residents, medical students identified many attributes, skills, and methods that led to a positive learning experience. The subthemes that arose most frequently and are, therefore, possibly most important to students include involving students in patient care, giving feedback, providing a safe environment, and being approachable. One might extrapolate from this that students want to feel engaged as part of a team of colleagues, that they wish to be integral to patient care, and that they crave the opportunity to learn through explicit and real-time evaluation. This echoes the situated learning theoretical framework whereby learners are fundamentally transformed as they collaborate with established community members—in this case, residents. Students acquire content expertise and community socialization through “legitimate peripheral participation” in germane work.^4^ The working relationships students have with more experienced members of a community of practice serve as the force that moves them from the periphery into the core of that specialty.

It is notable that most of the subthemes derived from this analysis focused on the performance of residents in terms of their facilitating work-based learning, the learning environment, and role modeling rather than focusing on dedicated time for formal teaching activities. This observation could provide encouragement to residents who feel they lack the time and expertise to teach medical students effectively. Educating clearly expands beyond a prepared lecture or chalk-talk, and this is generally, but not universally, recognized by medical students.

Despite the fact that most students recognized work-based teaching as part of the learning process, some students' evaluations did lament the scarcity of formalized discussion sessions. This highlights 2 important points: (1) Students crave protected time for education if residents are willing and able to provide it, and (2) some students may not recognize role modeling, supervision, and mentorship in the clinical setting as teaching. Some students may be conditioned by decades of schooling to think that lecture-based learning is the sine qua non of teaching, but this is not fully transferrable to clinical teaching. The same ingrained perceptions of what constitutes teaching may cause residents to discount their role in or the importance of work-based learning. It, therefore, behooves medical educators who supervise residents and medical students to ensure that both parties recognize the many flavors of teaching in the clinical environment. One mechanism to increase the perception that teaching is occurring could be the use of “signposting” to explicitly denote teaching points. This concept is already well established for identifying formative feedback which may be less easily recognized by trainees than summative feedback.^23^

Our hope is that the results of this study identifying the qualities, skills, and methods of effective resident teachers may be used to further develop more learner-informed RAT curricula. Such a curriculum could include only a few hours of programming, possibly during resident orientation, and ideally with refreshers throughout their training to reinforce key concepts. Subthemes belonging to themes 1 and 3 (“work based-learning, teaching, and assessment” and “learning environment”) appear most conducive to incorporating into a RAT curriculum (Table 2). Elements of theme 2 (“attitudes as teacher”) could be described to residents during their coursework but are felt less teachable. Theme 4 subthemes (“role modeling”) are things residents are already actively developing throughout their training but reminders to mind the example they set may be instructive. The content and context of teaching (themes 5 and 6) may be modifiers to teaching microskills covered from themes 1 and 3, as illustrated below.

**Table 2:** Suggested Simulation Scenarios and Skills Workshop Topics for a RAT Curriculum Informed by the Results of This Study

Subtheme	Suggested RAT simulation or skills workshop
1a. Sets expectations	“1st day on rotation” conversation
1b. Student involvement in care	Overseeing student calling consult Overseeing student updating family
1c. Student autonomy over care	Overseeing student performing physical examination Overseeing student-led clinical encounter
1d. Helps students prepare/practice	Student pre-rounds presentation practice Guiding student through interpretation of diagnostic data
1e. Gives feedback	Post-rotation summative feedback using the A.D.A.P.T. framework
1f. Mentorship and coaching	Post-encounter teaching using the 1-min preceptor model
1g. Challenges students	(Can make the learner in the scenarios for 1c more passive or shy)
3a. Psychological safety	Skills workshop on phrases and behaviors that promote safety
3b. Clear communication	(Concurrent with above)

Abbreviation: RAT = resident as teacher.

Themes 1 and 3 seem most conducive to resident education. Themes 5 and 6 may be modifiers to these scenarios. Theme 4 is outside the scope of RAT curricula. Theme 2 subthemes along with how to delegate responsibilities to students, the importance of signposting work-based learning and their function as role models should also explicitly be covered in RAT curricula.

We propose several examples of scenarios for role-playing simulations and topics for practice at skills workshops. Role-playing sessions and skills workshops may use 2 active residents (1 “resident-teacher” and 1 “student-learner”), observing resident(s), and a faculty facilitator. Resident actors could work through a scenario based on a scripted prompt followed by time for debriefing and feedback from all involved. Example scenarios include expectation setting (subtheme 1a) through a “first day of rotation” conversation, involving students in care (subtheme 1b) through overseeing a student calling a consult or updating family, allowing student autonomy over care (subtheme 1c) by overseeing a student-led encounter or physical examination, helping prepare (subtheme 1d) by practicing a patient presentation before rounds or guiding interpretation of diagnostic data, giving summative feedback (subtheme 1e) using the A.D.A.P.T. model,^24^ and providing mentorship or coaching (subtheme 1g) after a patient encounter using the 1-minute preceptor model.^25^ All of the above sessions would concurrently promote clear communication skills (subtheme 3b). Finally, a skills workshop to discuss and practice phrases and behaviors that promote psychological safety (subtheme 3a) would likely be of value.

In addition to these simulations and workshops, residents should be instructed to delegate responsibilities to the medical students they supervise. Residents should be practiced with identifying appropriate teaching cases for medical students, validating student contributions to patient care and decision making, and deciding which tasks would be acceptable for students to take the lead on (subthemes 1b/1c). Residents should be advised to be explicit about learning opportunities that arise in the context of their work and to “signpost” these lessons for their students. Residents should also be reminded of the power of the professional example they set for their students in role modeling positive and negative behaviors.

Notably, most themes derived from this analysis would seem to be generally applicable to resident teachers outside of neurology. The most neurology-specific comments tended to be in specialty-specific medical knowledge and skills (subthemes 5a and 5b). Therefore, RAT curricula across specialties may benefit from the lessons learned through this study.

Our findings are largely consistent with prior studies that analyzed student surveys, focus groups, and award nominations in other disciplines. All of these studies, including ours, found that superior residents were professional, supportive, friendly, and fun; they created a safe learning environment; and they included medical students in their work.^20,26-30^ Among trainees and faculty, the best clinical teachers for medical students and junior residents were described as being enthusiastic, clear and well-organized, and adept at interacting with learners independent of teaching method, professional role, or department.^31^ Of interest, such interpersonal elements enhance the quality of education more so than amount of teaching alone^10^ and 2 studies suggested that the learning environment or resident charisma were both more important than the clinical skill of the teacher.^32,33^ This is echoed in a review of resident evaluations of faculty teaching which found that there may be twice as many critical “noncognitive” aspects of teaching (creating a supportive learning environment, communication skills, and enthusiasm) as “cognitive” ones (clinical knowledge and skills).^34^ The perceived importance of expectation setting and feedback were inconsistent across these studies—although both were prominent subthemes in our analysis.

Many of the different paradigms for medical education learning theory include a tripartite interaction between the learner, the material to be learned, and the environment in which that learning is situated.^35^ Applying the themes derived from this analysis, one can see how resident teachers influence each of these 3 nodes, as well as the relationships binding them. Residents transform students through feedback (subtheme 1e) and mentorship (subtheme 1f). Residents select the material to be learned directly (theme 5) or indirectly by involving students in (subtheme 1b) and granting autonomy over patient care (subtheme 1c). This may even be subconscious as students strive to emulate their role models (theme 4). Residents create the learning environment by promoting safety (subtheme 3a) and clarity of communication (subtheme 3b). Residents modify the interaction between learner and material by challenging students (subtheme 1g). Residents modify the interaction between learner and environment by setting expectations (subtheme 1a) and helping them prepare or practice (subtheme 1d). Finally, residents' attitudes as teachers (theme 2) modulate the relationship between the material to be learned and the environment. Residents' influence on this paradigm can be felt across different contexts of teaching (theme 6).

Whereas previous studies have largely focused on faculty teachers from teacher or peer perspectives, this study focuses on neurology resident teachers of medical students from the student perspective. One strength of this work is that evaluations across 9 years were analyzed, which would reduce the effect of outside influences such as the changing of clerkship directors or the disruption of the COVID-19 pandemic. Another strength is that by using clerkship evaluation forms, the results are likely more representative of actual clinical experience than an experimental design may yield because of biases of the observer effect and the artificial scenario effect. It is of importance that the open-ended prompt used by evaluation forms contained no leading language to bias responses positively or negatively or directing them to comment on any specific features of interest to the authors. This study design also likely did not suffer from response bias, whereby strong or polarized opinions are over-represented because narrative comments were required from all students before submission.

Our work has several limitations. We were not able to include evaluations of former residents who remain at this institution for fellowship or faculty positions because of technical issues with the learning management system. It is conceivable that residents who stay at the same academic institution may teach or behave differently than those who leave. This study design is also only able to capture resident actions in practice at this institution and may, therefore, miss better practices in use elsewhere. Another limitation of this study is that by using open-ended evaluations, it is reliant upon respondents to recognize, recall, and report upon all important themes without specific prompting. A more specific prompt may have generated additional themes, especially negative ones.

Another potential significant but abstract limitation to this work is the relative lack of negative comments. With insufficient negative themes to contrast against the positive ones, it may be difficult to appreciate the most important teaching practices. There is another theoretical risk that students may have omitted negative comments out of fear of loss of anonymity. Educational practices (described in “Methods”) at this institution, however, minimize the risk of deidentification, so the authors believe this to be a negligible source of bias. Finally, student evaluations of residents may be biased by the “halo effect” whereby teachers who are more well liked for interpersonal reasons are evaluated more favorably.^34^

With respect to reflexivity, author H.W.S.'s status as a resident may have contributed in unconscious ways to their coding of deidentified student evaluations. It is felt that any bias this may have introduced was likely mitigated by double-coding with faculty author S.M.S. and by author J.J.M.'s review of the credibility of proposed codes.

Future directions for this research may include medical student focus groups to comment on the perceived validity of the findings of this study. Focus groups could also be used to more directly solicit medical student attitudes and expectations regarding resident teachers, as an extension to this study's indirect approach. Outpatient resident teaching practices could also be assessed in a more dedicated way with future study.

Much teaching done by residents is not “formalized” and occurs through the experience of clinical work in addition to resident role modeling behaviors. Our results support concepts in the existing literature and build upon them by focusing specifically on the resident-student relationships in the clinical setting. This provides additional perspective on how resident teachers fit into and affect the situated learning framework of learning theory. Our hope is that understanding this paradigm may help with continued RAT curricula design including the incorporation of instruction and simulations dedicated to communication and supervision of students as part of an active clinical work environment.

## Appendix Authors

**Table:**

Name	Location	Contribution
Harry W. Sutherland, MD	Department of Neurology, Yale School of Medicine, New Haven, CT	Drafting/revision of the manuscript for content, including medical writing for content; major role in the acquisition of data; study concept or design; analysis or interpretation of data
Jeremy J. Moeller, MD, MSc, FRCPC	Department of Neurology, Yale School of Medicine, New Haven, CT	Drafting/revision of the manuscript for content, including medical writing for content; study concept or design; analysis or interpretation of data
Sara M. Schaefer, MD, MHS, FAAN	Department of Neurology, Yale School of Medicine, New Haven, CT	Drafting/revision of the manuscript for content, including medical writing for content; major role in the acquisition of data; study concept or design; analysis or interpretation of data

## Glossary

COVID-19: coronavirus disease 2019
RAT: resident as teacher
VA: Veterans Affairs
